# Complement catalyzing glomerular diseases

**DOI:** 10.1007/s00441-021-03485-w

**Published:** 2021-10-06

**Authors:** Peter F. Zipfel, Thorsten Wiech, Hermann-Josef Gröne, Christine Skerka

**Affiliations:** 1 Department of Infection Biology, Leibniz Institute for Natural Product Research and Infection Biology, Jena, Germany; 2 Friedrich-Schiller-University, Jena, Germany; 3 Institute of Pathology, University Hospital Hamburg-Eppendorf, Hamburg, Germany; 4 Institute of Pharmacology, Philipps-University, Marburg, Germany

**Keywords:** Complement, Glomerular diseases, C3 glomerulopathy, Thrombotic microangiopathy, Complement diagnostics, C3 Convertase testing

## Abstract

Complement is an evolutionarily conserved system which is important in the defense against microorganisms and also in the elimination of modified or necrotic elements of the body. Complement is activated in a cascade type manner and activation and all steps of cascade progression are tightly controlled and regulatory interleaved with many processes of inflammatory machinery. Overshooting of the complement system due to dysregulation can result in the two prototypes of primary complement mediated renal diseases: C3 glomerulopathy and thrombotic microangiopathy. Apart from these, complement also is highly activated in many other inflammatory native kidney diseases, such as membranous nephropathy, ANCA-associated necrotizing glomerulonephritis, and IgA nephropathy. Moreover, it likely plays an important role also in the transplant setting, such as in antibody-mediated rejection or in hematopoietic stem cell transplant associated thrombotic microangiopathy. In this review, these glomerular disorders are discussed with regard to the role of complement in their pathogenesis. The consequential, respective clinical trials for complement inhibitory therapy strategies for these diseases are described.

## Introduction

Complement is an evolutionarily conserved system forming the central pillar of innate immunity. Complement acts at the early stage of an immune response and as a primary effector system can be initiated within seconds. This cascade upon activation generates effector compounds which recognize, handle, and can eliminate modified self-cells and also infectious microbes. The newly formed activation fragments orchestrate the infiltration and action of innate immune cells and attract adaptive immune cells and thereby modulate also adaptive immune function in a significant manner (Zipfel and Skerka 2009; Hajishengalis et al. 2017).

Properly activated complement maintains homeostasis and can induce a beneficial tightly adjusted inflammatory response. However, when deregulated, complement can induce acute or chronic inflammation which results in glomerular damage and in disease (Coulthard and Woodruff 2015; Laumonnier et al. 2017). The activated system directs toxic activation compounds to foreign or to modified target surfaces. Simultaneously, on self surfaces and intact host cells, activation and binding of such compounds is controlled and blocked. Tight control of this cascade at each step and regulation of each enzyme is therefore quintessential to avoid tissue and organ damage (Holers 2014).

Complement can adjust the strength of the inflammatory processes, and being involved in acute and chronic inflammation, tight regulation is required during initiation, effector function, and furthermore for adjusting the strength and the duration of the inflammatory response.

Inappropriate complement action in the kidney can be caused by self-reacting antibodies, by genetic, chromosomal alterations or by gene mutations affecting genes which encode single complement components (Bomback et al. 2016; McCullough et al. 2013). Glomerular diseases, with genetic or autoimmune causes affecting complement include C3 glomerulopathy (Zipfel et al. 2010a), hemolytic uremic syndrome (HUS) (Nester et al. 2015; Karpman et al. 2017), transplant associated thrombotic microangiopathy (TA-TMA) (Laskin et al. 2011), antibody-mediated transplant rejection (ABMR) (Stites et al. 2015), membranous nephropathy (Reinhard et al. 2021; Ronco and Debiec 2020), anti-neutrophil cytoplasmic antibody mediated vasculitis (ANCA) (Jayne 2019), and IgA nephropathy (IgAN) (Rauen et al. 2016; Rodriguez et al. 2017). Deregulated complement activation plays a major role in other pathologies or other renal diseases, which however cannot be discussed in detail in this review. Such diseases include post-/parainfectious endocapillary glomerulonephritis or autoimmune forms of interstitial nephritis.

**Fig. 1:**
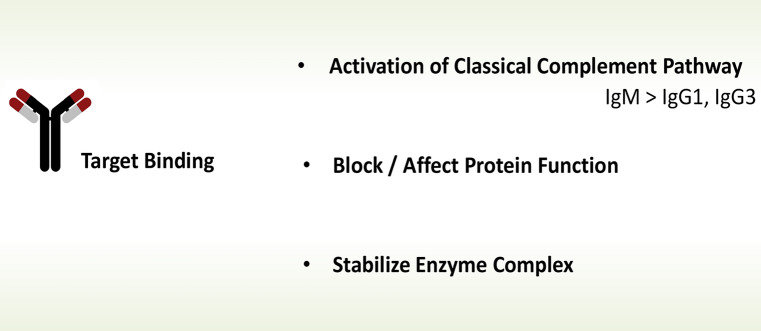
Different action of autoantibodies in glomerular diseases. Autoantibodies can influence complement by at least three mechanisms. Immune complexes activate the classical complement pathway. For this step the antibodies subtype is relevant. Autoantibodies of the IgM, IgG1, and IgG3 subtype activate the classical pathway of complement. However, IgE and IgA antibodies do not, or activate complement to a much lesser extent. Autoantibodies binding to single complement proteins or protein complexes influence protein function. The influence on protein function depends on the type of autoantigen, region to which the antibody binds and can cause different pathologies. In DEAP-HUS most autoantibodies bind to the C-terminal recognition region of Factor H and block Factor H surface binding. In C3 glomerulopathy autoantibodies bind to an anther region, the N-terminal regulatory region of the Factor H protein. Autoantibodies can also bind to neoepitopes in assembled protein complexes, like the AP C3 convertase C3bBb stabilized by properdin. In C3 glomerulopathy, many autoantibodies bind to neoepitopes of the alternative pathway C3 convertase, C3bBb and affect the stability of the enzymatic complex, block access of stabilizers or destabilizers and thereby alter the half-life of the active enzyme. Other antibodies bind directly to C3b, to Factor H or to Factor B. Thereby the antibodies influence the activity of this central complement activating enzyme in multiple ways

*Autoantibodies* in glomerular disorders can act in three ways. First, autoantibodies, upon target binding C1q bind to the autoantigen complex, becomes activated, recruits the proteases C1r and C1s, and activates the classical complement pathway. Second, autoantibodies bind directly to single complement proteins or enzymatic complexes and thereby affect/interfere directly with complement function. Third autoantibodies can bind to neoepitopes exposed on protein complexes like the convertase which represent active enzymes and thereby affect half-life, stability, or access of regulators (Fig. 1) (Wang et al. 2016, Zhao et al. 2019).

Identifying for each complement catalyzed glomerular disorder, the autoantibodies and the target of the antibodies allows to follow the mode of antibody action in detail. This can show which specific complement reaction is defective and often allows to localize the affected glomerular compartment. Such understanding is important to describe the exact pathophysiology, to improve diagnostics and is also relevant for precision therapy. Mapping the defective steps within the complement landscape likely will in future help to select the appropriate complement inhibitor(s) (Person et al. 2020). Furthermore, a detailed knowledge of the affected parameters allows to monitor the response of individual patients to the complement inhibitors, autoantibody therapy, and upon kidney transplantation.

*Genetic variations* in complement catalyzed glomerular disorders can result from chromosomal alterations, affecting several or single genes, or from mutations in single genes. Chromosomal alterations can result in hybrid or mutant proteins with duplicated protein domains. In addition, gene variations can cause exchange of single amino acids, can induce premature stop codons, or can induce a shift in the reading frame. Even rare genetic polymorphisms in complement genes can increase or decrease the risk for disease development, disease severity, or disease progression (Köttgen and Kiryluk 2021; Zipfel et al. 2020).

## Complement: initiation, enzymatic checkpoints, and effector function

**Fig. 2:**
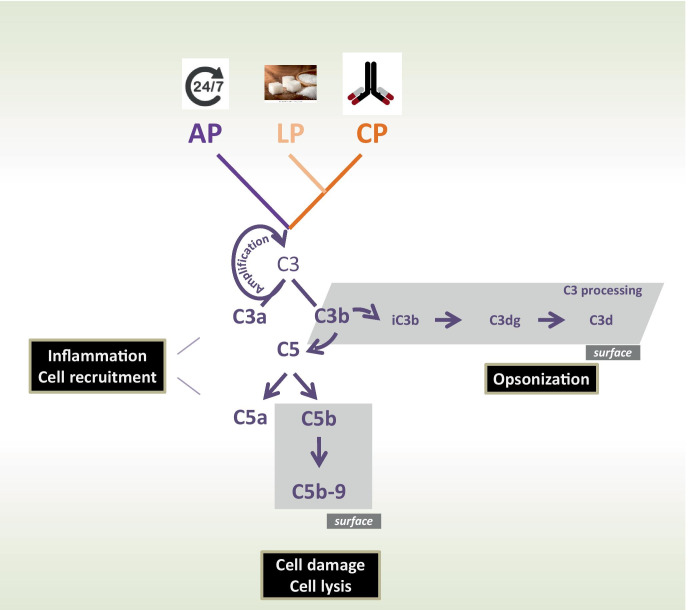
Complement activation and effector levels. The complement cascade is activated via three separate pathways: the alternative pathway (AP), the lectin pathway (LP), or the classical pathway (CP). The AP is activated by default, for 24 h, all day long and at a low level and in default setting further progression is blocked by inhibitors. The “decision” activation vs inhibition by the many regulators or inhibitors aims for activation on target surfaces. The AP pathway also forms a potent amplification loop at the C3 convertase level. The LP in initiated broadly speaking by carbohydrates and the CP by antibodies and immune complexes. The three pathways form C3 convertases that target C3 the central component of complement. C3 is activated and cleaved to C3a and C3b. C3a as anaphylatoxin drives inflammation and cell recruitment, C3b makes the fluid phase, surface transition and upon deposition on surfaces can form a surface bound C3 convertase. C3b deposition on target surfaces is termed opsonization and prepares the target for interaction and uptake by professional phagocytes. Deposited C3b can be processed further. C3b is inactivated to C3b (iC3b), and then processed to fragments C3dg or C3d. The processed forms of C3 can interact with specific cell receptors. Alternatively, C3b on the surface can form a C3 convertase, be involved in the amplification loop or trigger the conversion of a C3 convertase to a C5 convertases. This new enzyme C3bBbC3b targets C5 and splits C5 into the soluble anaphylatoxin C5a and the surface bound C5b. C5a drives inflammatory complement and C5b deposited on the target surface attracts the plasma proteins C6, C7, C8, and C9, thus allows assembly the C5b-9 and polymeric C9 which generate the terminal complex and the lytic pore

Complement is activated via three pathways, the alternative, the lectin, and the classical pathway, which trigger two major enzymatic checkpoints, the C3 convertase and the C5 convertase. These enzymes generate effector compounds, including the anaphylatoxins C3a and C5a, allow surface opsonization with C3b, and form the lytic terminal complement complex (TCC, also termed C5b-9). An overview how the complement cascade can progress and branch is presented in Fig. 2. This presentation further shows at which level the major effector compounds are formed allows to integrate the central effectors within the activated system and shows how they contribute to balanced homeotic regulation (Fig. 2).

Each major enzymatic level is directed by two distinct protein complexes, generated upon alternative or lectin/classical pathway activation, resulting in the C3 convertases of the alternative pathway, i.e., the C3bBb complex, or the lectin/classical pathway convertase, the C4bC2b complex. On this C3 convertase level, a highly potent amplification loop is activated which propels C3 conversion by the alternative pathway enzymes. The next level also forms two different C5 convertases, the alternative pathway convertase C3bBbC3b and the lectin/classical pathway convertase C4bC2bC3b. Both enzymatic convertases use plasma-derived C3 or C5 as substrate, and together with the amplification loop represent the central checkpoints for control. At present two activators which favor the formation of convertases are known. Properdin was already identified in 1954, and the second activator FHR5 (Factor H related protein 5) was identified in 2014 (Pillemer et al. 1954; Chen et al. 2016). In addition, multiple regulators, acting as inhibitors, regulators, or modulators, influence the various enzymatic reactions. Inhibitors, regulators, or modulators can be integral membrane proteins, and each membrane bound regulator has a unique cell expression profile. In addition, a large set of complement inhibitors are found in body fluids and in plasma, acting in solution, in fluid phase and they can reach most sites in the human organism. Interestingly plasma distributed regulators can also attach to surfaces. For example, both plasma regulators, Factor H and FHR1, bind to the glomerular basement membrane (Skerka et al. 2020).

### Fluid phase vs surface complement action

Complement activation of the alternative pathway initiates in the fluid phase and converts to surfaces. Ultimately, complement effector function is a surface event. Thus, many or even all effector steps of complement include a fluid phase to surface transition.

The central checkpoints of fluid phase vs surface transition and self vs non-self-discrimination include the following: assembly of the convertases of the alternative pathway in the fluid phase and transition to the appropriate target surface, as well as multiple regulated steps, which decide on cascade progression or on branch decision. In this regard, the type of response, amount of surface deposited C3b, processing of C3b, as well as a progression from the level of the C3 convertase to that of the C5 convertase are all tightly adjusted. The amplification loop of the alternative pathway adjusts the amount of the C3 convertase which are formed on target surfaces.

The C3 convertases and the C5 convertases are surface bound enzymes, which bind the soluble substrates, i.e., C3 or C5, and generate soluble anaphylatoxins (C3a, C5a) and surface bound C3b or C5b. C3a and C5a are released to the circulation, and ultimately, these anaphylatoxins bind to the corresponding receptors, which are expressed on the surface of target cells. Surface bound C3b, as well as C5b, induce specific effector functions. C3b or the processed fragments iC3b, C3dg, and C3d when recognized by specific receptors on target cells induce cell activation and trigger cellular effector functions, such as phagocytosis. Furthermore, antigen bound C3d can act as molecular adjuvant. Surface bound C5b attracts the soluble proteins, C6, C7, C8, and C9, and ultimately forms a pore, i.e., C5b-9.

Complement has two activators, i.e., properdin and FHR5, and multiple plasma-derived inhibitors, regulators, or modulators, including Factor H, FHL1, C4BP, FHR1, FHR2, FHR3, and FHR4 and several complement proteases, such as Factor I, C2, C1s, and C1r. Thus, complement is activated in the fluid phase and on surfaces, and the transition from fluid phase to the target surface is one central step for complement regulation and physiology (Zipfel and Skerka 2009). The activated complement cascade can follow different effector routes. Based also on the highly toxic effects, the inflammatory and cell damaging potential, all steps of these reactions are tightly controlled and regulated. The intensity of each step, i.e., initiation, cascade progression, and specific effectors is adjusted.

## Complement catalyzed glomerular diseases

Defective complement regulation causes glomerular pathology. Understanding how different autoimmune factors or genetic alterations influence the complement pathways, or pathway progression allows to understand pathophysiology, provides a rational for diagnosis and also for targeted interference in activation, blockage of specific levels, or to redirect deregulated pathway progression (Poppelaars and Thurman 2020).

### C3 glomerulopathy

C3 glomerulopathy is an umbrella term describing a group of related forms of glomerulonephritis, and dense deposit diseases, defined by dominant or exclusive glomerular C3 deposition usually detected by immunofluorescence or immunohistochemistry (Zipfel et al. 2010a; Pickering et al. 2013). Glomerular C3b deposition is explained by enhanced or defective complement regulation in the fluid phase. C3b deposition occurring at the glomerular basement membrane and on cell surfaces often progresses to terminal complement and results in the deposition of C5b-9. Standard diagnosis of C3 glomerulopathy is made by evaluating a kidney biopsy for intense C3 staining, substantially higher (two orders of magnitude) as immunoglobulin staining. Known autoimmune causes of C3 glomerulopathy include autoantibodies that target either single complement proteins or protein complexes (the C3 and the C5 convertase). In addition, chromosomal alterations, gene modifications, and gene mutations are reported in C3 glomerulopathy patients and families (Table 1). However, for many patients, the exact etiologic factors are still unknown.

**Table 1:** Autoimmune and genetic causes of glomerular complement catalyzed diseases

Disease	Autoimmune		Genetic		
**C3 Glomerulopathy**					
	**C3 Nephritic Factor**		***Factor H***	***FHR1***	
	**C5 Nephritic Factor**		**C3**	***FHR2***	
	**C4 Nephritic Factor**		***Factor I***	***FHR3***	
	**αFactor H**	**αC3**	***Factor B***	***FHR4***	
	**αFactor B**	**αC3b**		***FHR5***	
**DEAP-HUS / gHUS**					
	**αFactor H**		***Factor H***	***Factor I***	
		^**C−terminus**^	***C3***	***Factor B***	
			***MCP***	***DAGe***	
			***ΔΔFHR1-FHR3***		
**TA—TMA**					
	**αFactor H**				
		^**???**^			
**Membranous Nephropathy**					
	**αPLA2R**	**αFactor H**			
	**αTHSD7A**	^**C−terminus**^			
**ANCA**	αMPO				
	αMP				
**IgA Nephropathy**			***FHR1-FHR3***		***FHR5***
	**αΔgalactose-IgA1**		***ΔΔFHR1-FHR3* for Transplant**		

Autoimmune and genetic causes are linked to various glomerular diseases. Mutations and various affect the genes *Factor H, FHR1, FHR2, FHR3, FHR3, FHR4, FHR5, C3, Factor I*, and *Factor B*. In C3 glomerulopathy, autoimmune causes include immunoglobulins to C3 convertase, i.e., C3 nephritic factor, C4 nephritic factor, C5 nephritic factor, as well as autoantibodies targeting single proteins, i.e., Factor H, Factor B, C3, and C3b. In *DEAP-HUS*, autoimmune causes include antibodies which bind to the C-terminal region of Factor H. In DEAP-HUS patients, most antibodies develop on a genetic background of homozygous FHR1-FHR3 deficiency. Genetic causes of *HUS* affect the genes *Factor H, FHR1, FHR3, C3, Factor B, Factor I, MCP/CD46*, and *DAGe.* In *transplant-associated TMA (TA-TMA)*, autoantibodies to Factor H have been reported. Similarly, in *membranous nephropathy*, antibodies to Factor H are described. In addition antibodies to PLAR2 and to THSD7a are more frequent. *For ANCA*, antibodies to MPO and to proteinase 3 are reported. ANCA is a complement-mediated disease, and complement inhibition on the level of C5a and C5aR1 is efficient in clinical trials. *IgA nephropathy* are correlated with anti D-galactose-deficient IgA which deposits in the kidney. FHR1 and FHR3 homozygous deficiency has a strong protective role in IgA nephropathy, and *FHR5* is an IgA susceptibility gene. Elevated FHR1 levels correlate with severity of IgA nephropathy

Complement activation, leading to generation and subendothelial deposition of complement split products in cases of C3 glomerulopathy, mostly leads to the influx of macrophages and neutrophils, likely attracted by C3a and C5a. This leads to endocapillary hypercellularity, hence in early stages as typically seen in postinfectious, endocapillary glomerulonephritis (so-called atypical postinfectious glomerulonephritis) (Sethi et al. 2013). Ongoing, chronic and still sublytic complement action with additional deposition in the mesangium results in proliferation of mesangial cells (mesangioproliferative glomerulonephritis), and later to matrix increase in the mesangium and in the subendothelial space, leading to double contours and a membranoproliferative pattern. The exact characterization of the causes of complement activation is relevant to understand disease pathophysiology and pathology and to dissect which part of the complement cascade is deregulated and not acting properly. Such knowledge may allow to define new (plasma) biomarkers to stratify patient cohorts for optimal therapeutic regimen.

*Autoimmune factors* in C3 glomerulopathy include C3-Nef (C3-nephritic factor) which are autoantibodies that bind to neoepitopes of the assembled AP C3 convertase. C3Nef stabilizes the enzymatic C3bBb complex, followed by enhanced enzyme action which causes C3 turnover and can lead to C3 consumption. In addition, C4-convertase (C4-Nef), as well as C5-convertase (C5-Nef), targeting autoantibodies are described in C3 glomerulopathy (Corvillo et al. 2019; Noris et al. 2019; Marinzoni et al. 2017; Smith et al. 2019; Zhao et al. 2019; Zipfel et al. 2020). Such C3, C4, and C5 convertase-binding antibodies enhance the enzymatic action of the convertases, which form the central hubs of complement. Other pathologic autoantibodies target single complement proteins, such as Factor H, C3 activation products, i.e., C3b, iC3b, C3dg, C3d, and Factor B.

*Genetic causes of C3 glomerulopathy* have been reported and single complement genes, including the *Factor H, FHR1, FHR2, FHR3, FHR4, FHR5*, and the *C3* gene. Chromosomal alterations in the *FHR* gene cluster include deletions or duplications and often result in genes with have duplicated exons or in hybrid *FHR* genes. Such chromosomal alterations affect all five *FHR5* genes, but in these scenarios, the *Factor H* gene remained intact. Thus, disease develops due to defective or altered FHR proteins, modified FHR plasma levels, in presence of intact Factor H and FHL1 (Goodhship et al. 2017; Zipfel et al. 2010a; Zipfel et al. 2020). Also, *C3* and *Factor B* gene mutations are reported in C3 glomerulopathy. The effect of C3 mutations and how they cause pathology have been elaborated in elegant studies showing that the conversion of C3 in the formation of the C3 convertase complex is affected (Tortajada et al. 2013).

**Fig. 3:**
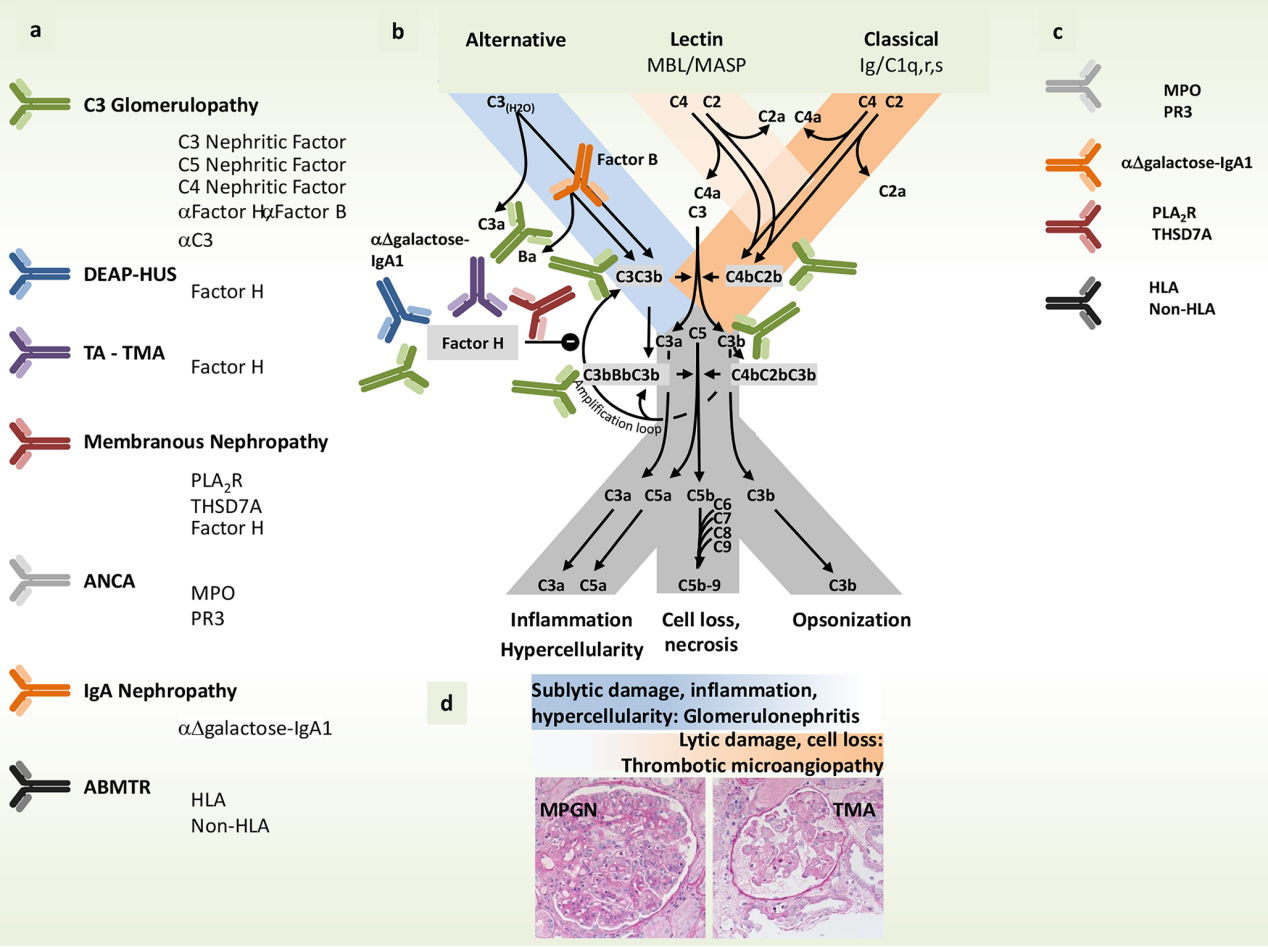
Targets of autoantibodies and genetic variations in complement catalyzed glomerular Diseases. **a** Several complement catalyzed glomerular diseases have autoimmune and genetic causes. These autoantibodies bind to multiple complement components and in some cases also attach to non-complement targets. Similarly, many complement genes and in some cases also non complement genes are altered in the diseases. The affected targets include complement convertases, convertase components and in particular various regulators. **b** Integration of the target components of the various autoantibodies in the complement cascade. **c** Targets of autoantibodies in glomerular disorders which are not directly linked to complement. **d** The spectrum of tissue reaction patterns to local complement activity: sublytic damage with proliferation and influx of inflammatory cells lead to the picture of membranoproliferative glomerulonephritis (MPGN, left), whereas lytic damage lead to the loss of endothelial cells followed by thrombus formation and the picture of **t**hrombotic **m**icro**a**ngiopathy (TMA, right)

Thus C3 glomerulopathy is caused by autoantibodies and genetic variations. The disease-related autoantibodies show heterogeneous binding profiles, and the genetic variations affect different complement genes. Therefore, multiple autoimmune and genetic scenarios can result in identical or highly related pathologies. The spectral forms and in particular the missing diagnostic tools to correlate biomarkers with the different forms of this glomerular disorder ask for additional and more precise diagnostic parameters (Fig. 3).

### HUS

Hemolytic uremic syndrome (HUS) is a complement disease with diverse etiologies, autoimmune, and/or genetic causes and can also be induced by infectious microbes. HUS is defined by hemolytic anemia, thrombocytopenia, and acute thrombotic microangiopathy (TMA), affecting primarily preglomerular arterioles in the kidney. Microthombi cause destruction and fragmentation of red blood cells, leading to thrombocytopenia and anemia with fragmentocytes. The common final path is damage of endothelial cells, followed by the formation of fibrin and platelet thrombi (TMA). The majority of HUS cases are caused by either shiga toxin producing, enterohemorrhagic, *Escherichia coli* (EHEC*)*, inducing Shigatoxin HUS (STEC-HUS), or by the Gram-positive bacterium *Streptococcus pneumoniae* inducing pneumococcal HUS. STEC-HUS and pneumococcal HUS are frequent in children (Westra et al. 2010; Meinel et al. 2018; Scobell et al. 2020). (Table 1).

#### Autoimmune factors in HUS

The autoimmune form of aHUS include antibodies which bind to the C-terminal recognition region of Factor H. This form which is termed DEAP-HUS (DEficient for FHR1-FHR3 and Factor H Autoantibody Positive) affects about 10 to 15% of HUS patients (Zipfel et al. 2010a, b; Jozsi et al. 2008; Martin Merinero et al. 2021; Pesce et al. 2020). Most DEAP-HUS patients present autoantibodies in context with a homozygous deletion of a 24 kb chromosomal segment that encompasses the *FHR3-FHR1* genes. In general terms DEAP-HUS affects young patients (ca 4–17 years of age) and most autoantibodies bind to the C-terminal recognition region of Factor H (SCRs19-20) and block Factor H surface binding.

#### Genetic causes of HUS

Genes mutated in HUS include *C3* and *Factor B*, which encode the proteins which form the alternative pathway C3 convertase (C3bBb). Other affected genes code for plasma regulators which adjust the activity of the C3 convertase, including Factor H, FHR1, FHR3, Factor I, thrombomodulin or for the membrane bound regulator MCP (membrane cofactor protein)/CD46. Thus, the mutated genes code for proteins which either form the AP C3-convertase or for regulators which adjust the action of this central complement enzyme. Proper AP C3 convertase activity on the endothelial surface is central for kidney homeostasis and alterations or deregulated action result in pathogenesis. In addition, also the cytoplasmic signaling protein Diacyl glycerol kinase epsilon (DAGKe) is linked to aHUS. DAGKe mutations might affect intracellular signaling within the endothelial cells in response to a complement trigger. The genetic causes of aHUS often show incomplete penetrance. For each gene mutation identified or for genetics variant it is always important to describe the pathogenic mechanisms and define how the mutant or the variant protein interferes in complement action and causes pathology.

The causative pathogenic factors in HUS are identified in many, but not in all patients. The genetic forms of HUS account for ca 15% of total cases and are more frequent in adults. In addition, autoimmune forms are found in children and adolescent patients.

### TA-TMA

Hematopoietic stem cell transplant associated thrombotic microangiopathy (TA-TMA) shows morphologic and histological features of TMA, similar to that in HUS, sharing microangiopathic hemolytic anemia, damaged platelets, and hemolysis. Similar to autoimmune DEAP-HUS, Factor H autoantibodies have been described in TA-TMA. In a small fraction of TA-TMA patients (ca. 3%), autoantibodies are described which were associated with heterozygous FHR3-FHR1 or FHR1-FHR4 deletion. The binding region of the antibodies need to be mapped in the Factor H protein in order to show that TA-TMA and DEAP-HUS antibodies show related binding profiles and cause related mechanisms for complement mediated endothelial damage and platelet activation. Furthermore, it would be of interest to compare the autoantibodie titers and duration of the autoantibodies response in TA-TMA patients with that of DEAP-HUS.

### Membranous nephropathy

Membranous nephropathy is a kidney disease with circulating autoantibodies targeting autoantigens expressed on the surface of podocytes. It is assumed that immune complexes that form at the glomerular basement membrane activate the complement system. Ultimately, the terminal complement complexes (TCC or C5b-9) lead to podocyte damage and induce alterations of the basement membrane and defective barrier function disturbs glomerular filtration and can result in proteinuria (Meyer-Schwesinger et al. 2015; Tomas et al. 2014; Haddad et al. 2019, Reinhard et al. 2021).

Primary membranous nephropathy is mediated by autoantibodies to M-type phosphoplipase A_2_ receptor (PLA_2_R) (95%) and rarely to thrombospondin type 1 domain containing 7a (THSD7a) receptor, a podocyte antigen (3–5%). Recently, additional autoimmune forms were described, where autoantibodies developed which target complement Factor H (3%), exostosin 1 or exostosin 2 (Seikrit et al. 2018; Sethi et al. 2019). The presence of Factor H antibodies and the role of such autoantibodies for pathology of membranous nephropathy is controversial (Valoti et al. 2019). Factor H autoantibodies were not identified in all MN cohorts and the presence during disease course can vary.

Most PLA_2_R and THSD7A autoantibodies are of the IgG4 subtype; thus, an immunoglobulin subtype, which shows minimal effect on alternative complement pathway activation. However, also other autoantibody subtypes (IgG1, IgG2, and IgG3) with the ability to activate complement via the classical pathway have been identified (Tomas et al. 2014, 2016; von Haxthausen et al. 2018). Components of the classical and the alternative pathways are prominently localized at the site of the IgG-antigen deposits. Also, lectin pathway activation is considered to initiate complement in membranous nephropathy (Haddad et al. 2019). The IgG-antigen complexes can be found along Jayne et al. 2017 the outside of the GBM at the anchoring side of the podocyte foot processes (Zipfel et al. 2020). The GBM matrix expands, grows and disease develops. Immunhistochemistry and mass spectrometry from in kidney biopsies from patients with PLA_2_R1 associated membranous nephropathy show deposition of complement compounds, including C3b and C5b-9. Thus, complement activation can likely be initiated by antibodies which bind to PLA_2_R1 or THSD7a and upon activation complement C5b-9 is deposited, likely causing sublytic podocyte damage, leading to the retraction of foot processes, the loss of slit diaphragms and the development of nephrotic proteinuria.

### ANCA-associated glomerulonephritis

ANCA (anti-neutrophil cytoplasmic antibody)-associated vasculitis (AAV) encompasses related diseases, including granulomatosis with polyangiitis (GPA, Wegeners granulomatosis), microscopic polyangiitis (MPA), and eosinophilic granulomatosis with polyangiitis (EGPA; or Churg-Strauss syndrome). Damage is induced in small blood vessels of the kidney, but also, other organs, such as lungs, nerves, and sinuses, are affected. A characteristic feature of the disease is the presence of autoantibodies which target the neutrophil myeloperoxidase (MPO) or the proteinase 3 (PK3) (Brogdan and Eleftheriou 2018). In addition, the alternative pathway of complement likely is activated, leading to complement mediated inflammation (Wester et al. 2019). Newly generated anaphylatoxin C5a attracts immune cells, especially neutrophil granulocytes and monocytes. In consequence, a vicious inflammatory cycle is induced: more neutrophils are attracted to the local sites and the cells when activated release their granular content. These reactions in the microcirculation and in glomerular capillaries can lead to ruptures of the glomerular basement membrane, a step which is synonymous with necrosis. This is followed by proliferation of parietal cells, together with infiltrated macrophages forming crescents, which over time become more and more sclerotic.

### IgA nephropathy

IgA nephropathy (IgAN) as a leading cause of chronic kidney diseases is likely made up of several subgroups with different pathogenetic factors (Zhou et al. 2021). Glomerular deposition of galactose-deficient IgA1 immune complexes may trigger complement activation via the alternative pathway, resulting in deposition of complement components. Genome-wide association studies linked the *FHR*-gene cluster with IgA Nephropathy (Gharavi et al. 2011; Zhu et al. 2018; Zhou et al. 2021). Homozygous deletion of *FHR1-FHR3* has a strong protective role. In addition, elevated FHR1 plasma levels and pathogenic variants of Factor H are associated with pathology and higher FHR1 plasma levels are reported in patients with disease progression Ref (Roman et al. 2017).

Based on elevated FHR1 plasma levels it was suggested that more FHR1, and less Factor H is bound at damaged sites (Tortajada et al. 2019). Thereby, enhanced FHR1 triggered inflammation and stronger C3 fragment deposition due to lower inhibition via Factor H can impair renal function. Other *FHR* genes are also linked to this kidney disease, and opposing effects were reported (Skerka et al. 2020; Zipfel et al. 2020). Homozygous FHR1/FHR3 deficiency is protective and FHR*5* gene variations cause pathology (Xie et al. 2016, 2020; Sanchez Rodriguez et al. 2020; Dragon-Durey et al. 2017; Medjeral-Thomas et al. 2017a, b). *FHR5* gene variants contribute to disease susceptibility (Zhai et al. 2016). The FHR5 variants showed altered binding to C3b and three recombinant mutant proteins bound C3b with higher intensity. Thus, FHR5, the complement activator, can bind to damaged tissues via deposited C3b and also via newly exposed laminin (Rudnick et al. 2018).

In addition to the alternative pathway, also the lectin pathway is linked to IgAN (Maillard et al. 2015). Serum levels of mannan-binding lectin (MBL-2) vary in IgA patients (Guo et al. 2017), rare genetic variants in the MBL2 gene increase the risk of progression for IgAN (Ouyang et al. 2019), and deposition of MBL and L-ficolin is identified in histological lesions of IgAN patients (Roose et al. 2006).

### Antibody-mediated renal transplant rejection

One of the most relevant complications after allogenic kidney transplantation is antibody-mediated rejection (ABMR). Allelic variations of human leukocyte antigens (HLAs) class I or II can lead to a mismatch between donor and recipient followed by recognition as a foreign molecule with the development of donor-specific antibodies (DSAs) (Bhalla et al. 2020). When these DSAs bind to the HLA on the endothelial cells of the graft, several immune mechanisms are initiated and attract inflammatory cells, resulting in the histopathological picture of glomerulitis and peritubular capillaritis. One relevant mechanism seems to be the initiation of the complement system via the classical pathway (Stites et al. 2015) since it has been shown that in patients with DSA that bind to C1q death-censored ABMR-free and allograft survivals were significantly lower (Bamoulid et al. 2017). Since not only ABMR but also ischemia/reperfusion injury leads to complement activation after organ transplantation, complement inhibition has been tried to improve graft survival. Recently a phase 2, open-label single-arm trial demonstrated the efficacy of eculizumab along with thymoglobulin induction in preventing acute ABMR in transplanted kidneys (Glotz et al. 2019).

## Novel diagnostic tools for precision complement monitoring

The current standard technique to assess the involvement of the complement system in kidney biopsies is immunofluorescence or immunohistochemistry. For combined with morphological alterations the diagnosis of glomerulonephritis, most renal pathologists stain routinely for C3c, some also for C1q, C4d, and C5b-9, in addition to immunoglobulin A, IgG, and IgM. The result is an individual staining pattern that provides evidence for the pathogenesis. Dominant positivity for IgA is seen in IgA nephropathy, a “full house” positivity for all markers is typically seen in lupus nephritis, and dominant or exclusive positivity for C3c and C5b-9 is observed in C3 glomerulopathy. In many cases, this approach leads to a sufficient distinction. There are, however, two major disadvantages of these methods: (a) larger molecules, especially IgM and C1q, but maybe also C3c to a certain extent, can be unspecifically trapped in the glomerular matrix pretending immune complex deposition and complement activation, and (b) the staining patterns reflect accumulation of deposited immunoglobulins and complement factors within the glomeruli without clear correlation with the activity of immunological events.

**Fig. 4:**
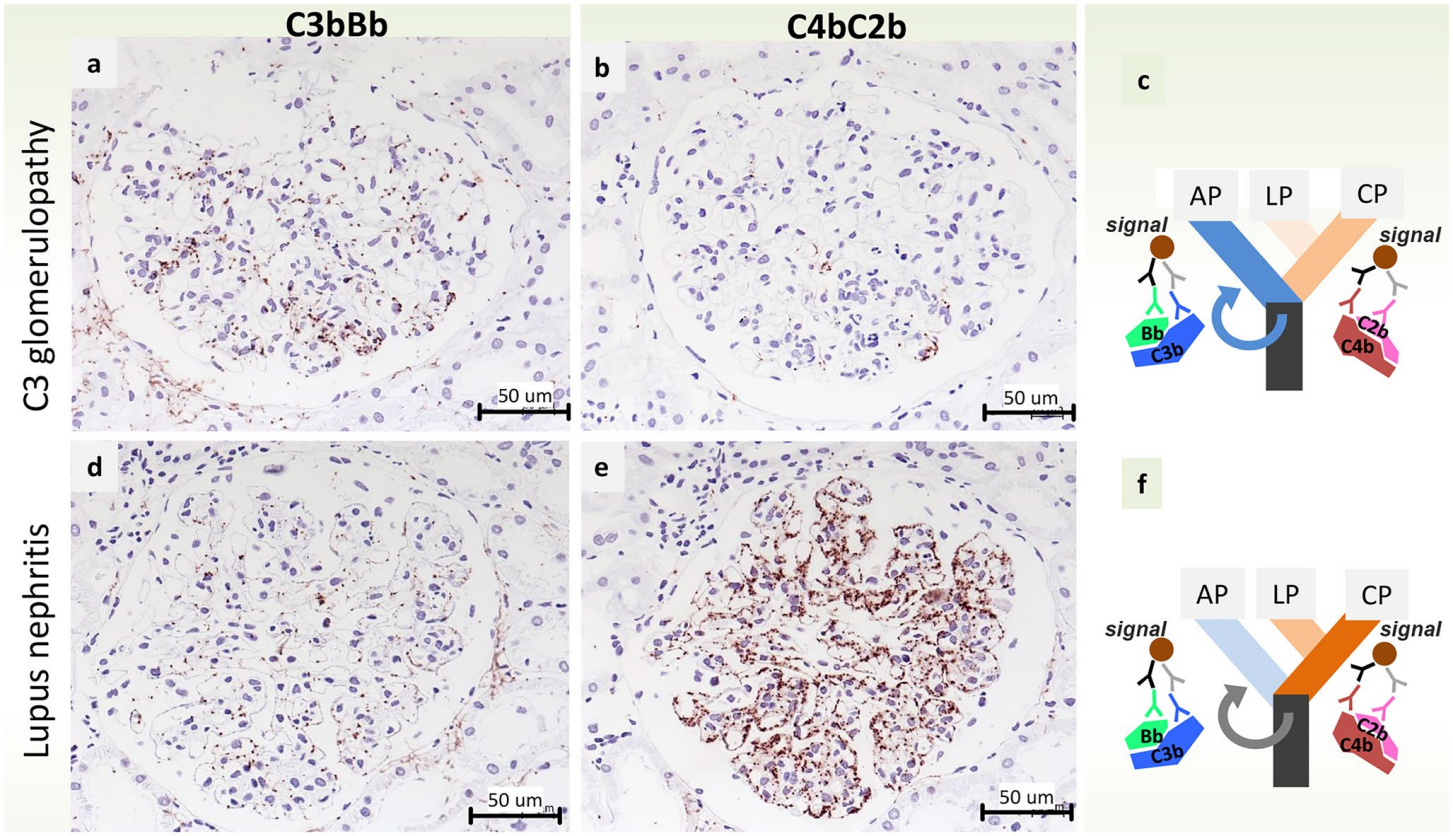
Alternative vs classical complement pathway activation in C3 glomerulopathy and in systemic lupus erythematosus. In situ visualization of complement convertases using proximity ligation assays detecting proximity of the single components reveal high densities of signals (brown dots) for the alternative convertase C3bBb in C3 glomerulopathy (**a**), but only very few signals for the classical/lectin convertase C4bC2b (**b**). In contrast, in lupus nephritis (class IV) few signals for C3bBb (**d**), but a very high signal density for C4bC2b (**e**) is seen. These results indicate, as expected for these disorders, a predominant activation of the alternative complement pathway in C3 glomerulopathy (**c**) and a predominant activation of the classical pathway in lupus nephritis (**f**)

In order to provide novel precision tools to measure local complement activity in biopsies, we developed a methodological workflow to detect assembled—and therefore likely active—C3/C5 complement convertases in tissue (Person et al. 2020). We use brightfield proximity ligation assays for the detection of assembled C3b with the fragment Bb attached, the alternative convertase, and for C2b with C4b, the convertase of the classical/lectin pathway. The staining results can be easily located within the tissue and are easily quantified. Figure 4 shows an example of a C3 glomerulopathy with predominant assembly of alternative convertases, in contrast to a case of lupus nephritis (class IV) with predominant classical/lectin pathway convertases.

## General considerations, different mode of action of autoantibodies

Complement catalyzed glomerular disorders are multifactorial and can be caused by autoantibodies, by gene variations, or are triggered by infections. Three of seven complement-catalyzed glomerular diseases, discussed here, have both autoimmune and genetic causes, and multiple scenarios do exist. Different autoimmune factors and mutations in several complement genes can cause the same disease. In a reverse setting, even autoantibodies targeting the same protein or mutations in the same complement gene can cause pathology in several diseases. For even other diseases where autoimmune factors target noncomplement proteins, complement is involved in pathology and therapeutic complement inhibition is protective.

Autoantibodies targeting the plasma protein complement Factor H are reported in four glomerular diseases, C3 glomerulopathy, DEAP-HUS, TA-TMA, and membranous nephropathy. Similarly, mutations in the *Factor H, FHR1, FHR3, FHR5*, and *C3* genes are described in two or more diseases, such as C3 glomerulopathy, genetic HUS, IgA nephropathy, and AMD.

The pathologic effects in of autoimmune C3 glomerulopathy and DEAP-HUS are well characterized. In both disease autoantibodies bind Factor H, but these antibodies target different functional regions of Factor H and they bind or do not bind to FHL1, the truncated variant which is derived from an alternatively spliced transcript of the *Factor H* gene. In C3 glomerulopathy autoantibodies bind to the N-terminal regulatory region of Factor H and FHL-1 and they block fluid phase regulation of both plasma proteins. In DEAP-HUS, in contrast, the majority of antibodies bind to the C terminal recognition region of Factor H and they do not bind to FHL1. These antibodies block Factor H surface binding exclusively. In both TA-TMA and in membranous nephropathy, the binding domains in Factor H and the pathologic role of the autoantibodies have not been characterized in detail, yet. The correlation of Factor H autoantibodies and homozygous FHR1-FHR3 deficiency exist in DEAP-HUS, but not in the other disorders.

These scenarios show multifactorial roles of single complement proteins. They also highlight the complex pathology of complement catalyzed glomerular disorders, showing that dysregulation of the complement network can occur at different levels and can result in the same disease. This demonstrates that regulation of the AP C3 convertase which forms one important hub in the complement cascade is tightly controlled. Furthermore, these results show important roles for the FHR1, FHR3, and FHR5 proteins.

In C3, glomerulopathy multiple autoimmune factors and alterations of several genes contribute to the same pathology. In HUS, one type of autoantibody is dominant and several genes, most of which code for complement proteins are affected. In IgA nephropathy, autoimmune forms exist, *FHR5* gene variations cause pathology and FHR1 plasma levels influence disease prognosis (Chen et al. 2016; Zhai et al. 2016).

In other glomerular diseases, antibodies bind to proteins, which so far are not linked to the complement system. However, the beneficial effect of complement inhibition in ANCA-associated necrotizing glomerulonephritis clearly shows that complement is involved in diseases pathology (Jayne 2019).

Broadly speaking, genetic mutations in genes which encode secreted plasma proteins can manifest in two manners: they can block protein secretion resulting in accumulation of the mutant proteins in the cytoplasm, or in other cases secretion is not affected, then mutant proteins are identified in plasma but protein function is altered (Ault et al. 1997). As genetic variations can be homo- or heterozygous, mutations result either in reduced or absent plasma proteins, or protein function is affected in 50% or in all proteins. Variations in the genes *Factor H, FHR1, FHR3*, and *C3* are reported in both C3 glomerulopathy and in HUS. Furthermore, instability of the FHR gene cluster can result in hybrid genes, encoding hybrid FHR::FHR proteins, as well as Factor H::FHR hybrid proteins, or in *FHR* genes with duplicated exons and may result in mutant FHR proteins that have duplicated SCR domains (Groopman et al. 2016). These variations in the *FHR* gene cluster and mutations in the *FHR* encoding genes in several glomerular disorders confirm that each single FHR protein, i.e., FHR1, FHR2, FHR3, FHR4 and FHR5 represents a major complement and immune regulator, e.g., FHR1 being an inflammatory mediator or competitor of Factor H in surface binding, or FHR5 as a complement activator propels cascade progression (Skerka et al. 2020; Chen et al. 2016; Irmscher et al. 2019).

Autoimmune causes or genetic alterations can ultimately result in the same pathology and in the same kidney disease. Thus, showing the alternative pathway is central for several glomerular disorders and multiple scenarios can alter the action of the AP C3 convertases of complement. Many autoimmune and genetic alterations affect either the components which form the AP C3 convertase, or they alter the regulators, which adjust the activity of this central enzyme or they influence regulator portfolio at local sites. The effect of the C5a and C5aR1 targeting compounds Vilobelimab (IFX-1) and Avacopan (CCX168) demonstrate the relevance of the inflammatory axis of complement in glomerular diseases. Thus based on autoimmune and genetic results show that even in the same disease different targets or steps/levels of the cascade can be affected (Khalili et al. 2020).

## Complement inhibitors

Given the clear association of various autoimmune and genetic causes in glomerular diseases, it is clear that complement inhibition is monitored for these disorders. The inhibitors being evaluated in clinical studies of various complement catalyzed glomerular disorders are summarized in Table 2.

**Table 2:** Clinical trials with complement inhibitors in complement catalyzed diseases

Compound	Name	Target	Company	C3G		aHUS	Thrombotic Microangio-pathies	TA-TMA	Membranous Nephropathy	ANCA		IgA Nephropathy	ABMR		PNH
**OMS721**	**Narsoplimab**	**MASP-2**	**Omeros**	**C3G**		**aHUS**	**Thrombotic Microangiopathies**			**SLE**	**IgA Nephropahty**				
**APL2**	**Pegcetacoplan**	**C3**	**Apellis**	**Glomerulopathies**											**PNH**
**LNP023**	**Iptacopan**	**Factor B**	**Novartis**	**C3G**					**Membranous Nephropathy**			**IgA Nephropahty**			**PNH**
				transplanted and non transplanted											
**CCX168**	**Avacopan**	**C5aR1**	**Chemocentryx**	**C3G**		**aHUS**				**ANCA**		**IgA Nephropathy**			
	**Eculizumab**	**C5**	**Alexion**					**TA-TMA**					**ABMR**		
	**Ravalizumab**	**C5**													
**C5 Antibody**	**Pozelimab**	**C5**	**Regeneron**												
**IFX-1**	**Vilobelimab**	**C5a**	**InflaRx**							**ANCA**					
**ACH-0144471**	**Danicopan**	**Factor D**	**Achillion/Alexion**	**MPGN**											
**TP10**	**soluble CR1**	**CR1**													
**ALN-CC5**	**Cemdisiran**	**C5 mRNA**	**Alnylam**			**aHUS**			**Membranous Nephropathy**			**IgA Nephropahty**			
	small interfering RNA														
**Coversin**	**rVA576**		**Akari**							**pediatric HST-TMA**					
**Berinert**	**C1 esterase**		**CSL Behring**										**ABMR**		
**CPV-101**	**recFactor H**		**Eleva**		**C3G**										

Complement inhibitors which act in different levels and steps of the cascade are being evaluated in clinical trials. Source NIH clinical trials; https://www.clinicaltrials.gov

### Complement inhibitors for C3 glomerulopathy

Complement inhibition in C3 glomerulopathy using Eculizumab was tested and a group of patients, but not all patients responded to this terminal pathway inhibitor (Schubart et al. 2010). Additional complement inhibitors being tested include OMS721/Narsoplimab directed to MASP2 (Omeros), LNP023/Iptacopan targeting Factor B (Novartis), APL2/Pegcetaplocan directed to C3 (Apellis), ACH014447/Danicopan targeting Factor D (Achillion Alexion), IFX1/Vilobelimab targeting C5a (InflaRx), CCX168/Avacopan addressing C5aR1 (Chemocentryx), ALN-CC5/Cemdisiran a small interfering RNA inhibiting C5 production by the liver (Avacopan) and Coversin /rVA576/Nomacopan a tick (Ornithodros moubata) interacting with C5 and directly binding to C5d, CUB and C5C345C domains of C5 (Akari Therapeutics) recombinant moss produced Factor H, (eleva). Studies with TP10, a soluble CR1 derivative in C3 glomerulopathy are stopped.

### Complement inhibitors for HUS

Complement inhibition by Eculizumab/Soliris and just recently by the long-acting inhibitor Ravulizumab/ Ultomiris (Alexion) is approved for genetic, atypical HUS (Legendre et al. 2013; Rondeau et al. 2020). Additional complement inhibitors tested in this disease include OMS721/Narsoplimab, CCX168/Avacopan (Chemocentryx) Vifor Pharmaceuticals. ALN-CC5/Ccx168/Cemdisiran (Avacopan) and Coversin /rVA576/Nomacopan (Akari Therapeutics, https://www.akaritx.com/).

### Complement inhibitors evaluated in TA-TMA

Complement blockade by Eculizumab is used to treat pediatric and adult patients with TA-TMA (Gavriilaki and Brodsky 2020). Additional complement inhibitors are evaluated for treatment of TA-TMA and phase II studies with the Lectin pathway MASP-2 inhibitor OMS721/Narsoplimab and in pediatric HSC-TMA patient with the tick derived complement inhibitor coversin (rVA576) are ongoing (Akari Therapeutics)(web page).

### Complement inhibitors in membranous nephropathy

Ongoing clinical studies for *membranous nephropathy* include the complement inhibitors OMS-721/Narsoplimab, which targets the lectin pathway protease MASP2, LNP023/Iptacopan the Factor B inhibitor and Coversin /rVA576/Nomacopan.

### Clinical trials with complement inhibitors in ANCA

In ANCA, known autoantibodies target the noncomplement protein proteinase K and myeloperoxidase. However, complement inhibition at the C5a::C5aR1 axis with IFX1 and CCX168/avacopan (Inflarx, Chemocentryx) has protective effects. In addition, other C5 targeting inhibitors like ALN-CC5/cemdisiran, the interfering RNA blocking C5 production (avacopan) and coversin /rVA576/nomacopan the tick derived inhibitor (Akari Therapeutics) are used in clinical trials.

### Clinical trials with complement inhibitors in IgA nephropathy

Complement is associated with IgA nephropathy, but the exact mechanism and initiation pathway is not defined so far. Complement inhibition in IgA nephropathy is moved forward with the inhibitors OMS721/narsoplimab, LNP023/iptacopan, APL-2/pegcetacoplan, and at the C5 level, the C5 targeting small interfering RNA cemdisiran, ravulizumab, coversin/rVA576/nomacopan, and CCX168/avacopan (Lafayette et al. 2020; Selvaskandan et al. 2020).

### Clinical trials with antibody-mediated transplant rejection

For prevention of delayed graft function (NCT01919346) and in the therapy for chronic complement-mediated injury in kidney transplantation (NCT01327573) complement inhibition using eculizumab/soliris has been investigated. For prevention of rejection, a C1-esterase inhibitor (Berinert®, NCT01134510) will be evaluated.

This large number of inhibitors being evaluated in these complement catalyzing glomerular diseases and which block the complement cascade at different levels or adjust different effector pathways give promising options for the efficient therapy of these diverse glomerular disorders.

## Outlook and perspective

Complement defects cause several glomerular disorders. Understanding which part of the complement pathway is deregulated or which effector step is defective is highly relevant for both diagnosis, therapy and also to predict success of kidney translants. A detailed evaluation of complement parameters both in plasma and in renal biopsies is one requisite for precision diagnostic to understand which part of complement is deregulated, to the exact pathology for each single patient and to interfere with defective complement action. Such detailed evaluation of the complement plasma profile in combination with an elaborate kidney diagnosis is relevant to identify the primary pathologic features of complement associated glomerular diseases. Given the large list of complement next generation inhibitors and the growing numbers of tools to identify exactly the underlying complement alterations and the pathophysiological mechanisms in individual patients more and more successful therapy of these complement catalyzed glomerular disorders can be expected.
